# Early Experience with Reduction of Unstable Pelvic Fracture Using a Computer-Aided Reduction Frame

**DOI:** 10.1155/2018/7297635

**Published:** 2018-02-13

**Authors:** Jing-Xin Zhao, Li-Cheng Zhang, Xiu-Yun Su, Zhe Zhao, Yan-Peng Zhao, Guo-Fei Sun, Li-Hai Zhang, Pei-Fu Tang

**Affiliations:** ^1^Department of Orthopaedics, Chinese PLA General Hospital, No. 28 Fuxing Road, Haidian District, Beijing 100853, China; ^2^Department of Orthopaedics, Beijing Tsinghua Changgung Hospital, School of Clinical Medicine, Tsinghua University, No. 168 Li Tang Road, Changping District, Beijing 102218, China

## Abstract

**Purpose:**

The optimal closed reduction technique for unstable pelvic fractures remains controversial. The purpose of this study is to verify the effectiveness and report early experiences with the reduction of unstable pelvic fractures using a computer-aided pelvic reduction frame.

**Methods:**

From January 2015 to August 2016, a total of 10 patients with unilateral unstable pelvic fractures were included in this study. The surgical reduction procedure was based on the protocol of the computer-aided pelvic reduction frame that we proposed in a previous work. The quality of the reductions achieved using this system was evaluated with residual translational and rotational differences between the actual and virtual reduction positions of pelvis. The duration of the operation was recorded for quality control.

**Results:**

The mean times required to set up the frame, to complete the virtual surgery simulation, and to reduce the unstable pelvic fractures were 10.3, 20.9, and 7.5 min, respectively. The maximum residual translational and rotational displacements were less than 6.5 mm and 3.71 degrees, respectively.

**Conclusions:**

This computer-aided reduction frame can be a useful tool for the speedy and accurate reduction of unstable pelvic fractures. Further clinical studies should be conducted with larger patient samples to verify its safety and efficacy.

## 1. Introduction

Pelvic fractures, especially unstable pelvic fractures, are associated with massive hemorrhage and injury to important organs, and they can cause significant morbidity and mortality. Early reduction and fixation have the advantages of better pain relief, early mobilization, easier nursing care, and improved bleeding control [1–3], and they have come to be associated with lower morbidity, shorter ICU stays, fewer transfusions, and lower rates of complications such as in-hospital infection, thromboembolism, and pressure ulcers [1]. They may also be capable of preventing the long-term complications of posterior pelvic ring fracture, such as malunion, which eventually lead to differences in the lengths of the lower extremities and the resulting lower back pain.

However, the optimal early reduction technique for unstable pelvic fractures remains unclear. There are currently two types of reduction techniques, mainly classified as open and closed. Because extensive open surgery for pelvic fracture is usually associated with increased bleeding, increased risk of neurovascular injury, and the potential of a second hit to the hemodynamically unstable trauma patients, there has been a growing trend toward using different types of external fixators to reduce the unstable pelvic fracture and displacement through various types of lower extremity traction and intraoperative temporary stabilization, followed by definitive fixation of percutaneous iliosacral screws with or without additional anterior fixations. This is called a closed procedure.

The classic external fixator for the unstable pelvic fracture reduction is called the pelvic C-clamp. It was designed in the 1990s [4], and it is associated with several major intraoperative complications related to the superior gluteal artery and nerve. Since then, several types of external fixators have been designed to reduce the unstable pelvic fractures by manipulating the anterior pelvic ring. Bellabarba et al. placed two Schanz screws to the anterior inferior iliac spine (AIIS) and used a single external fixation bar to secure them for use as levers to reduce the external rotation of the affected hemipelvis in lateral compression (LC) pelvic fractures [5]. This technique has been revised and verified in the following clinical and biomechanical studies [6–8]. Sellei et al. designed a special X-frame to try to provide more posterior pelvic compression for reduction manipulation [9]. Queipo-De-Llano et al. used a pretensed curved bar as a means to reduce unstable pelvic fractures by applying a simultaneous compression on the posterior and anterior rings [10].

With these tools, the reduction position of affected hemipelvis had to be maintained manually until definitive fixation was complete. To overcome this problem, Matta and Yerasimides [11] and Lefaivre et al. [12, 13] each designed another type of pelvic reduction frames to connect the intact and injured hemipelvis to the operating table to perform the reduction manipulations in a stepwise manner. Inspired by the configuration of the Starr pelvic reduction frame designed by Lefaivre et al. [12, 13], we developed a computer-aided reduction mechanism for unstable pelvic fractures. The hardware of the entire system is based on three remote center of motion (RCM) mechanisms articulated with each other, and the software incorporates the 3-dimensional (3D) reconstruction pelvic model based on the intraoperative CT data, matrix algorithms procedure, and several commercial computer-aided design (CAD) software packages, which are used to perform the virtual reduction operations. In our previous study [14], the precision of the entire system was established and verified, including the rotational and translational precision for different degrees of freedom. From January 2015 to August 2016, we used this system to treat 10 patients with unstable pelvic fractures. Here, we report the effectiveness and early experience with reduction of the unstable pelvic fractures using this computer-aided pelvic reduction frame.

As with the Starr pelvic reduction frame [12, 13], all the reduction manipulations of the displaced hemipelvis, including translational and rotational ones, are performed with the reference position of its intact counterpart, which has to be held securely to the operating table by the external frame. This can ensure that the reduction position by the reduction frame is equal to its simulated position calculated using CAD software. Thus, the indication of this system is limited to unilateral unstable pelvic fractures.

## 2. Materials and Methods

### 2.1. Patient Characteristics

The study was approved by the local ethics and institutional review committee and registered on the ISRCTN registry (registration number: ISRCTN38873803). Informed consent was obtained from all participants included in the study. From January 2015 to August 2016, a total of 10 patients admitted in our institution, including 7 males and 3 females with an average age of 41.5 years (range, 31 to 55 years), were selected and included in this clinical research. The mean Injury Severity Score was 29.5 (range, 17 to 53). The mean time from injury to surgery was 4.7 days (range, 4 h to 21 days). The indication of this pelvic reduction frame was the same as that of the Starr frame, which were the unilateral unstable pelvic fractures and displacements. The pelvic fractures were classified according to the Young-Burgess [15] and OTA classifications [16]. According to Young-Burgess classification, there were four LC II type fractures, four APC II type fractures, one APC III type fracture, and one VS type fracture. According to OTA classification, there were four 61-B1 fractures, four 61-B2 type fractures, and two 61-C1 type fractures.

### 2.2. Surgical Procedure

The entire frame consists of two large side arc bars, which are used to secure the intact hemipelvis, and two smaller side arc bars, which are used to connect and hold the injured hemipelvis. The actual position of the injured hemipelvis is controlled by a Schanz screw, also called the end-effector of the system, which can slide on the anterior arc bar and rotate around the center of the anterior arc. The anterior arc bar connects the bilateral smaller side arc bars and can slide on them. When the anterior arc bar slides on the smaller bilateral side arc bars, the end-effector can rotate around the center of the smaller side arc bar in the lateral view.

**Figure 1:**
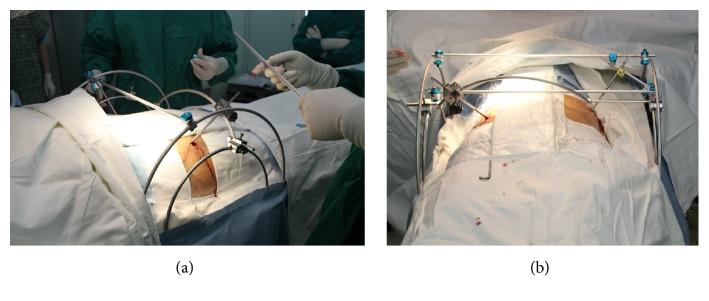
The frame configurations and setup during the operation.

During the reduction procedure, the patients were placed in supine position. The Schanz screws were connected with the larger side arc bars and placed in the intact hemipelvis. The end-effector Schanz screw was placed into the AIIS of the injured hemipelvis in the direction from AIIS to posterior superior iliac spine (PSIS), the so-called LC II screw (Figure 1).

**Figure 2:**
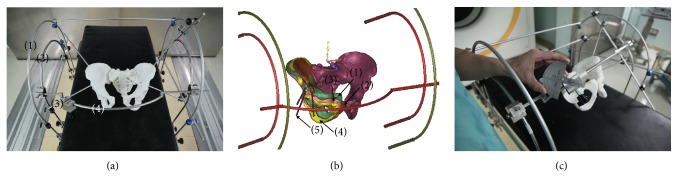
The frame configurations and reduction process were presented with the pelvic 3D printed and reconstruction models. (a) The frame configurations include two larger side arc bars (1), which are used for securing the intact hemipelvis, and two smaller side arc bars (2), which are used for connecting and holding the injured hemipelvis. The injured hemipelvis can be rotated or translated by the smaller side arc bars through the LC II screw (3) in a controlled manner. The rotations of hemipelvis can be performed around the center of the anterior arc on the anterior arc bar (4) in the LC II plane and the LC II screw in the plane vertical to LC II screw, and along the smaller side arc bars in the sagittal plane. (b) The reduction process of the rotational displacements of the injured hemipelvis can be explained by four hemipelves in four different virtual places. Hemipelves (1) and (2) are the hemipelves at displaced and reduction places, respectively. Hemipelves (3) and (4) are two intermediate places, which are rotated from hemipelves (1) and (2), respectively. The rotation degrees from hemipelvis (1) to (3) and hemipelvis (2) to (4) can be calculated by using the intersection degrees of their respective LC II screws in the sagittal and LC II planes, respectively. In this condition, there will be only one self-rotation around the LC II screw (5) with hemipelves (3) or (4), which are parallel to each other in space and can be calculated using the matrix transformation of 3D rotation around an arbitrary axis as follows:$\left[\begin{array}{ccc} n_{x}^{2}(1-\cos\theta )+\cos\theta & n_{y}n_{x}\left(1-\cos\theta \right)+n_{z}\sin\theta & n_{x}n_{z}\left(1-\cos\theta \right)-n_{y}\sin\theta \\ n_{y}n_{x}\left(1-\cos\theta \right)-n_{z}\sin\theta & n_{y}^{2}(1-\cos\theta )+\cos\theta & n_{z}n_{x}\left(1-\cos\theta \right)+n_{x}\sin\theta \\ n_{z}n_{x}\left(1-\cos\theta \right)+n_{y}\sin\theta & n_{z}n_{y}\left(1-\cos\theta \right)-n_{x}\sin\theta & n_{z}^{2}(1-\cos\theta )+\cos\theta \end{array}\right]$. (c) During the operation, the self-rotation around the LC II screw could be completed with a specialized protractor.

After assembly of the entire frame and positioning the patient, the patient and frame were processed with the intraoperative CT scan. Then, based on the intraoperative CT scan data, several commercial CAD software packages will be used to reconstruct the 3D models of the pelvis and frame and calculate the reduction process. In general, the entire reduction process can be broken down into rotations and translations in three directions, rotations around the center of the anterior arc on the anterior arc bar in the LC II plane and the LC II screw in the plane vertical to LC II screw, movement along the side arc bars in the sagittal plane, and cephalic, caudal, and lateral translations. The reduction process of rotational displacements was presented in Figure 2.

We then performed virtual reduction using CAD software. After each step in the reduction process and the virtual final reduction position was verified by the software, the actual reduction manipulations of the unstable pelvic fracture will be performed with the reduction frame according to the calculation results. The details of the reduction functions of the entire frame are described in our previous publication [14].

For evaluation of the reduction quality of the clinical application of the entire system, these series of patients underwent a second intraoperative CT scan after reduction with the frame. Based on this second intraoperative CT scan data, the 3D pelvic model at the anatomical reduction position can be reconstructed. The residual translational and rotational differences between the actual and virtual anatomical reduction positions were calculated with the matrix transformation between two positions using CAD software. They represent the reduction quality of this frame. Based on the translational and rotational residues, we fine-tuned the reduction position and took the inlet and outlet views using C-arm to confirm the results before definite fixation.

In addition, the operation time was also recorded for the quality control of this technique.

### 2.3. Statistical Analysis

Before fine-tuning, the residual translational and rotational differences between the actual and virtual anatomical reduction positions in each direction were computed and compared with zero using one-sample Student's *t*-test.

## 3. Results

Because the entire frame consisted of several RCM mechanisms, bars, and specialized connectors, the setup process was a time-consuming process before the beginning of the operation. The mean time required for setting up the frame, the virtual surgery simulation, and the reduction of the unstable pelvic fractures was 10.3 min (range, 7.7–12.1 min), 20.9 min (range, 18.1–22.5 min), and 7.5 min (range, 6.3–9.9 min), respectively. No complications, including the nerve or vascular injures, were reported during the operations performed on this series of patients.

The reduction results are shown in Table 1. As shown, the average residual translational displacements in each direction were slightly larger than the rotational ones, which might indicate that the entire system could control the rotational displacements of the injured hemipelvis better than the translational displacements. The maximum residual translational displacement of the unstable pelvic fracture was less than 6.5 mm with an average value of 2.38 mm, and the maximum residual rotational displacement was less than 3.71 degrees with an average value of 1.55 degrees. These values indicate the accuracy of the whole system during the clinical applications. There were significant differences between the residual translational and rotational displacements and neutral position in each direction. However, the reduction position almost fulfilled the clinical requirements according to the previous published standards [17–19].

**Figure 3:**
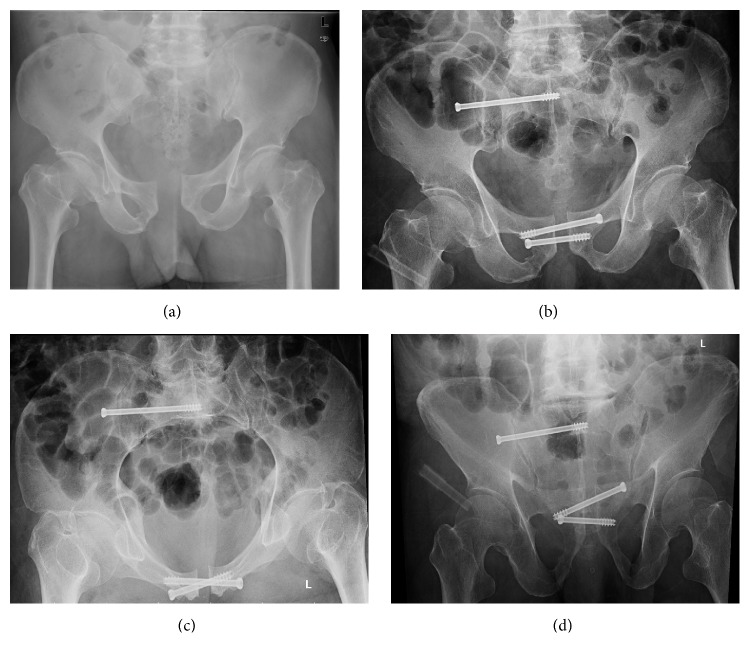
Case number 1. (a) Preoperative AP view radiograph; (b), (c), and (d) postoperative AP, inlet and outlet view radiographs.

**Figure 4:**
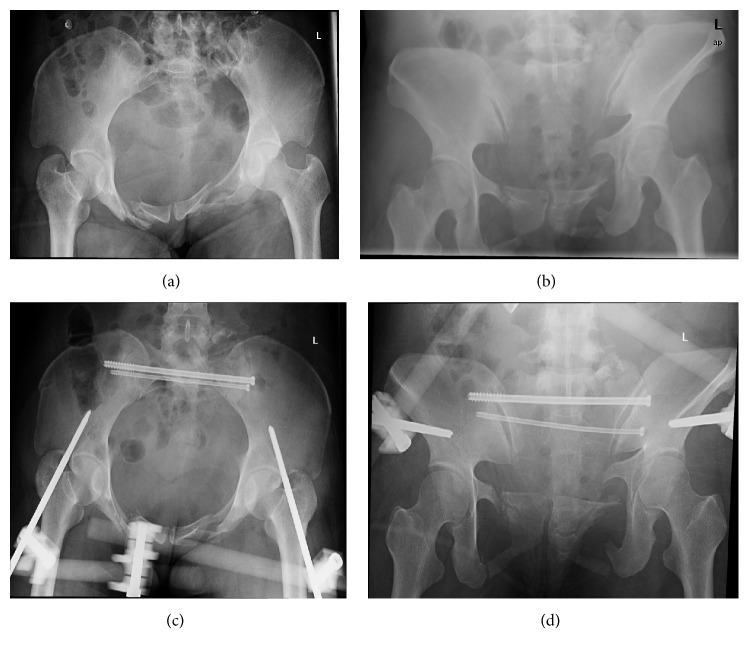
Case number 2. (a) and (b) Preoperative inlet and outlet view radiographs; (c) and (d) postoperative inlet and outlet view radiographs.

Figures 3 and 4 show two cases of clinical applications of the established frame and system.

## 4. Discussion

Regarding the closed reduction and percutaneous fixation of the unstable pelvic fractures, several methods and techniques have been proposed, including different patient positions, equipment for lower extremity traction, and stabilization of reduction positions of injured hemipelvis. Accurate reduction for the unstable pelvic fractures has been recognized as the cornerstone of safe placement of iliosacral screws, but no consensus has been reached regarding the optimal reduction technique.

Our previous study confirmed the accuracy of the system* in vitro*, which yielded maximum residual translational and rotational displacements less than 5 mm and 4 degrees, respectively; these values meet clinical requirements, and they can be classified as good according to Majeed [17] and excellent or nearly excellent according to Lindahl et al. [18] and Matta and Tornetta [19]. The present results indicate that the quality of rotational reduction was slightly better than that of the translational reductions. One possible reason for this might be that the entire system consisted of RCM mechanisms, the function of which was to hold the target in place and rotate it around the remote center of motion in a precise manner. However, the translational reduction is usually performed by lower extremity traction or moving the injured hemipelvis as a whole body controlled by the frame and Schanz screws, which inevitably produces uncontrolled errors.

The results of the present study show that, except for residual translation in the *z*-axis, the maximum residual translational displacement of the unstable pelvic fracture in the clinical applications was less than 4.6 mm, with an average value of 2.29 mm; and the maximum residual rotational displacement was less than 3.71 degrees, with an average value of 1.55 degrees. The residual rotational and translational displacements in each direction were greater than the corresponding values published in our previous study [14]. One reason for this may be the resistance from the elasticity of the soft tissue inside the pelvis. Another reason why residual translation in the *z*-axis was slightly greater than in other directions might be the design and manufacture of the frame, which was the same as that shown in our previous study [14]. Furthermore, the accuracy of the system could be improved with better configuration and manufacture of the frame, even though there is no standard or grading that can precisely quantify the residual rotational displacements of the hemipelvis in unstable pelvic fractures. Meanwhile, the elasticity of the soft tissue should be considered an important factor for better design, manufacturing, and experimentation in the next generation of reduction frames.

The time elapsed from injury to surgery is another important risk factor for the fracture reduction manipulation. It is easier for the close reduction manipulation of almost all suitable fractures in the initial phase of fractures. As time goes on, varying degrees of consolidation take place at the site of the fracture, increasing the difficulty of close reduction manipulations. Thus, the delayed unstable pelvic fractures with significant bony consolidation should be evaluated with caution beforehand to ensure the reduction quality of this technique.

The traditional reduction procedure for pelvic fractures is based primarily on stepwise manual maneuvers verified via intraoperative fluoroscopy. Most measurement methods used to verify traditional reduction results are also based on the 2D radiographs, which can only quantify the translational displacements and assess the quality of the rotational direction of the hemipelvis. A literature review by Mataliotakis and Giannoudis outlined the various measurement systems proposed by different authors [20]. The radiological evaluation of pelvic translational displacement depend mainly on the pelvic AP view [18, 19, 21, 22], the outlet view [23], and the inlet view [22]. Many authors argue that the current radiographic measurement methods used for fractured pelvises lack standardization, well-accepted reliability, and validity [13, 21].

Using our system, the measurement method was based on a 3D reconstruction model of the pelvis, which could indicate the translational and rotational displacements at the same time through calculation of the transformation matrix between the displaced hemipelvis and the reduced hemipelvis. Although no intra- or interobserver reliability studies of this method have been performed, the software and the equation itself should not cause any intra- or interobserver differences. Such differences are related primarily to the accuracy of the manufacture of the frame.

Because no single software program could meet the needs of this study, different software packages with different functions were used to perform the calculations and simulations at various points in the process. Mimics software (Materialise, Haasrode, Belgium) was used to construct the 3D model of the pelvis. The Geomagic software (Research Triangle Park, NC, USA) was used to calculate the transformation matrix of the hemipelves between locations. The 3-Matic software (Materialise) was used to perform the visual simulation of the reduction processes. Although many export and import steps were involved in the processes, the spatial orientation and coordinates of the pelvic models used in these software packages were consistent, and they served as the basis of the calculations used in this study. Thus, the accuracy of the system might be minimally influenced by the repetitive manipulations of several types of software. The total time required for the virtual manipulation was approximately 20.9 min.

Several types of CAD software had to be used in this study to complete the anatomical reduction of the unstable pelvic fracture using the frame. There might be a learning curve required before untrained surgeons can use these software packages, almost all of which are professional computer graphics programs. As given above, we have been developing professional software to expedite the process.

Because the authors have encountered difficulties with the closed reduction of unstable pelvic fractures, the procedure reported in this study was designed and implemented step by step. It was informed by knowledge of applied computer software, computer graphics, physics, computer navigation, and radiologic imaging. Many computer-assisted or robotic surgeries to reduce long bone fractures have been reported. A study of computer-assisted fracture reduction for pelvic fractures was reported in 2002 [24]; however, this procedure was still a real-time virtual operation performed by the surgeons using a registration method based on the preoperative CT dataset. The advantage of the present study was the use of an intraoperative CT-based registration method, which greatly improved the accuracy of registration and the subsequent virtual operation.

The overdose radiation problem encountered in clinical orthopedic trauma practices was the impetus underlying the development of this system. We attempted to combine the techniques of intraoperative imaging and virtual surgery to facilitate the performance of anatomical closed reductions using minimally invasive pelvic surgery. We believe that with development of computer-assisted surgery techniques and orthopedic surgery robots, radiation exposure could be eliminated or mitigated in the future. The RCM mechanism that is predominantly used in medical surgery robotic systems was the prototype for the final pelvic reduction frame presented in this study. Our team has been developing a reduction robot for use with long bone fractures, and we have used it in tibial fracture reduction, and the results have been published in research journals [25–29]. The algorithm and mechanism of the present system could also be the basis for the further studies on the pelvic fracture reduction robot.

As the indication of this technique is limited to unilateral unstable pelvic fractures, the number of patients included in this study was relatively small. The advantages and disadvantages of this technique should be evaluated with a larger number of participating patients and a matched control group of the traditional ORIF treatment with long-term follow-up.

## 5. Conclusions

This computer-aided reduction frame can be a useful tool for the speedy and accurate reduction of unstable pelvic fractures. Further clinical studies should be conducted with larger patient samples to verify its safety and efficacy.

## Figures and Tables

**Table 1:** The translational and rotational residual displacements in each direction.

Type	Axis	Measurement (*n* = 10, mean ± SD)	|Max|	|Min|	*T*	*P*
Translational (mm)	*X*	2.43 ± 1.2	4.6	0.98	6.404	0.0001
	*Y*	2.15 ± 0.95	3.58	0.73	7.157	0.0001
	*Z*	2.57 ± 2.1	6.5	0.21	6.252	0.0001

Rotational (degrees)	*X*	1.63 ± 1.05	3.29	0.01	10.56	0.0001
	*Y*	1.55 ± 0.64	2.51	0.42	7.659	0.0001
	*Z*	1.48 ± 1.3	3.71	0.07	3.6	0.0057
